# Transmission prevention behaviors in US households with SARS-CoV-2 cases in 2020

**DOI:** 10.3389/fepid.2023.1160214

**Published:** 2023-06-02

**Authors:** Rebecca J. Rubinstein, Wenwen Mei, Caitlin A. Cassidy, Gabrielle Streeter, Christopher Basham, Carla Cerami, Feng-Chang Lin, Jessica T. Lin, Katie R. Mollan

**Affiliations:** 1 Department of Epidemiology, University of North Carolina at Chapel Hill, Chapel Hill, NC, United States; 2 Department of Biostatistics, University of North Carolina at Chapel Hill, Chapel Hill, NC, United States; 3 College of Arts and Sciences, University of North Carolina at Chapel Hill, Chapel Hill, NC, United States; 4 Institute of Global Health and Infectious Diseases, School of Medicine, University of North Carolina at Chapel Hill, Chapel Hill, NC, United States; 5 Medical Research Council Unit, The Gambia, London School of Hygiene & Tropical Medicine, Banjul, Gambia; 6 Center for AIDS Research, School of Medicine, University of North Carolina at Chapel Hill, Chapel Hill, NC, United States

**Keywords:** COVID-19, household transmission, cohort studies, housing, health disparities

## Abstract

**Introduction:**

Severe acute respiratory syndrome coronavirus-2 (SARS-CoV-2) transmission frequently occurs within households, yet few studies describe which household contacts and household units are most likely to engage in transmission-interrupting behaviors.

**Methods:**

We analyzed a COVID-19 prospective household transmission cohort in North Carolina (April to October 2020) to quantify changes in physical distancing behaviors among household contacts over 14 days. We evaluated which household contacts were most likely to ever mask at home and to ever share a bedroom with the index case between days 7–14.

**Results:**

In the presence of a household COVID-19 infection, 24% of household contacts reported ever masking at home during the week before study entry. Masking in the home between days 7–14 was reported by 26% of household contacts and was more likely for participants who observed their household index case wearing a mask. Participants of color and participants in high-density households were more likely to mask at home. After adjusting for race/ethnicity, living density was not as clearly associated with masking. Symptomatic household contacts were more likely to share a bedroom with the index case. Working individuals and those with comorbidities avoided sharing a bedroom with the index case.

**Discussion:**

In-home masking during household exposure to COVID-19 was infrequent in 2020. In light of the ongoing transmission of SARS-CoV-2, these findings underscore a need for health campaigns to increase the feasibility and social desirability of in-home masking among exposed household members. Joint messaging on social responsibility and prevention of breakthrough infections, reinfections, and long COVID-19 may help motivate transmission-interruption behaviors.

## 1. Introduction

Households are a high-risk setting for the transmission of severe acute respiratory syndrome coronavirus-2 (SARS-CoV-2), especially when individuals positive for SARS-CoV-2 are unable to self-isolate. Infected individuals may face challenges distancing from family members and wearing masks at home, and they are unlikely to take precautions just before symptom onset, when viral shedding and infectiousness peak (1–4). In 2020, before widespread vaccination, high rates of household secondary attacks were identified in the United States, including a rate of 52% among households in Wisconsin and Tennessee and 60% in North Carolina (4, 5). A majority of secondary cases were identified within a week of the index case presenting symptoms (4, 5). Although vaccination greatly reduces the likelihood of severe disease, outbreaks of the more-transmissible Delta and Omicron variants and subvariants have occurred among vaccinated index cases and close contacts in households across the USA (6–8).

Modifiable risk factors to help interrupt household transmission include masking at home and avoiding sharing a bedroom with infected individuals (4, 8, 9). Previous studies support immediate isolation within one’s household upon testing positive (8). However, few published studies have characterized which household contacts and household units are most likely to engage in behaviors that interrupt transmission, and the structural barriers that can prevent them from doing so, including high household living density (10, 11).

The aims of the current study are as follows: (1) to describe changes in household contacts’ COVID-19 mitigating behaviors (e.g., mask-wearing, sharing a bedroom with the primary infected case) between cohort entry and day 14 of cohort participation; and (2) to identify structural and individual-level factors associated with these behaviors at day 14. We analyzed behavioral data from the COVID-19 Household Transmission Study (CO-HOST), a racially and ethnically diverse cohort of household transmission in central North Carolina conducted between April and October 2020, encompassing rural, suburban, and urban households (4). In 2020, both the original Wuhan strain of SARS-CoV-2 and the D614G “G” variant circulated across the USA (12). At that time, public health guidance recommended 14 days of self-quarantine after possible COVID exposure.

Our findings can help guide prevention efforts for the household transmission of SARS-CoV-2 in North Carolina and comparable regions. Given the frequency of novel and highly transmissible SARS-CoV-2 variants and challenges to herd immunity in the USA (13, 14), including vaccine hesitancy (15), a better understanding of the behaviors that contribute to preventing transmission in infected households can alleviate future waves of SARS-CoV-2 in the USA.

## 2 Materials and methods

### 2.1 Study sample and design

The CO-HOST study recruited patients infected with SARS-CoV-2 who sought care and tested at a UNC Respiratory Diagnostic Center in Chapel Hill, Cary, or Raleigh, NC (index cases). We recruited adults aged 18 years and over who tested positive for SARS-CoV-2 RNA with a qualitative nasopharyngeal swab polymerase chain reaction (PCR) test performed at the UNC hospital clinical laboratory. These adults were classified as index cases in the CO-HOST study. To participate, index cases had to willingly self-isolate at home for a 14-day period. Index cases who lived alone were not eligible to participate, as we required cases to live with at least one household member who was also willing to participate (herein referred to as household contacts). The inclusion of household contacts was limited to individuals aged 1 year and older, currently living in the same residence as the index case, with no plans to live elsewhere over the 28 days of study participation. The primary aim of the CO-HOST study was to determine the household secondary attack rate of SARS-CoV-2 infection in central North Carolina. Detailed inclusion criteria, follow-up testing, classification of index cases and household contacts, and study aims have been previously described (4). Ethical approval for the parent study was received from the Institutional Review Board at the University of North Carolina at Chapel Hill (Protocol No. 20–0982), participants gave informed consent before participating, and the parent study conformed to the principles outlined in the Declaration of Helsinki.

At cohort entry (day 0), along with PCR nasopharyngeal and saliva testing, we asked all index cases and household contacts whether they ever masked at home in the previous 7 days. The index cases and household contacts were also asked about COVID-19 symptoms, comorbidities, sociodemographic characteristics, and their activities in the previous 7 days. The index cases and household contacts completed electronic symptom diaries until 2 consecutive days without symptoms. Asymptomatic household contacts continued to complete the symptom diaries until day 21 to detect new symptoms. If participants missed ≥2 days of questionnaires, the symptoms were ascertained by study staff over the phone (4). On day 14, the household contacts again received testing and answered the same questions about symptoms and behaviors in the past 7 days (e.g., days 7–14 after cohort entry). In the present study, the analysis of exposures and outcomes was limited to household contacts.

### 2.2. Outcomes

Household contacts were asked whether they engaged in the following activities with the index case within the 7 days before cohort entry and between days 7 and 14: sharing a bedroom, sharing a bathroom, sharing a kitchen, watching television, eating together, sharing car rides, and sharing electronic devices. The primary behavioral outcomes for inferential analyses were as follows: (1) did the household contact ever wear a mask at home between days 7 and 14 (yes/no); and (2) did the household contact ever share a bedroom with the index case between days 7 and 14 (yes/no). Individuals were coded as having masked or shared a bedroom with the index case if they engaged in these behaviors on one or more occasions between days 7 and 14.

### 2.3. Exposures

We assessed the association of the following individual-level factors among household contacts to the outcomes: age, sex, race/ethnicity, and aged 50 years or older or reporting ≥1 comorbidity. We assessed age as a binary risk factor, comparing adults aged 50 years and older to those aged 18–49 years to reflect the non-linear increased risk of severe COVID-19 occurring in older adults (4, 16). We also asked the household contacts about the following factors on day 14, asking them to recall days 7–14: duration of COVID-19 symptoms, primary caregiving to the index case, and working outside the home. For each household contact, we assessed household-level exposures including high living density (>3 individuals in <6 rooms, including bedrooms, kitchen, and common rooms, but not bathrooms or garage) and whether the household contact observed the index case wearing a mask 7–14 days after cohort entry.

### 2.4. Statistical analysis

Changes in the proportion of household contacts engaged in shared behaviors with the index case over 14 days were estimated among the participants with non-missing responses. To account for clustering within households, we used the Yang modification of Obuchowski’s test for changes in paired binary data (17), executed in the clust.bin.pair package (v01.1.2) of R version 4.0.5 (18).

We estimated associations between exposure variables and household contacts (1) ever masking at home and (2) sharing a bedroom with the index case at day 14 using log-binomial models fit with generalized estimating equations to account for clustering of contacts within households (using Windows SAS 9.4). Prevalence ratios were estimated because of the cross-sectional nature of the analyses between exposures and outcomes between days 7 and 14 of cohort participation. Individuals were coded as having masked or shared a bedroom with the index case if they engaged in these behaviors on one or more occasions between days 7 and 14. For the exposures of COVID-19 symptom duration, primary caregiving to the index case, and working outside the home, we also asked participants to recall their participation in these activities between days 7 and 14. We did not access the frequency of engaging in these activities. It was impossible to establish temporality between some of the exposures and the masking and bedroom-sharing outcomes from these data. For each outcome, an intracluster correlation (ICC) with 95% confidence intervals (CI) was estimated using a linear mixed model calculated with the SAS ICC9 macro (19, 20). In the sensitivity analyses, missing data were handled using multiple imputation (MI) for clustered multilevel data, using the jomo package in R version 4.0.2 (21, 22). A type I error rate of alpha 0.05 was applied throughout, with no adjustment for multiplicity.

## 3. Results

Between April and October 2020, 100 households with 204 eligible household contacts were enrolled into the CO-HOST study (4). Two households and four household contacts were excluded due to incomplete study follow-up (Supplementary Figure S1). A majority of household contacts did not know their own infection status while answering surveys at cohort entry and day 14, although they were aware that the index case was infected at study entry. Despite not necessarily knowing their own infection status, over half (54%) of household contacts at cohort entry reported symptoms consistent with COVID-19 infection in the previous 7 days (Table 1).

CO-HOST household contacts were racially and ethnically diverse. Almost half (48%) of the participants self-identified as Black, Indigenous, or People of Color (BIPOC), including a high proportion of Hispanic/Latinx participants (34%). Of the participants, 23% resided in “high-density” households, with more than three people occupying fewer than six living spaces (Table 1). The median age was 26 years (range 1–85 years). Most participants (86%) lived with at least one other person at high risk of experiencing complications from COVID-19 infection, including individuals aged 50 years and older and those with obesity or comorbidities. Together, these characteristics illustrate a cohort of exposed household members vulnerable to the downstream effects of COVID-19 infection. Baseline characteristics are shown separately for BIPOC and White non-Hispanic participants (Table 1).

**FIGURE 1:**
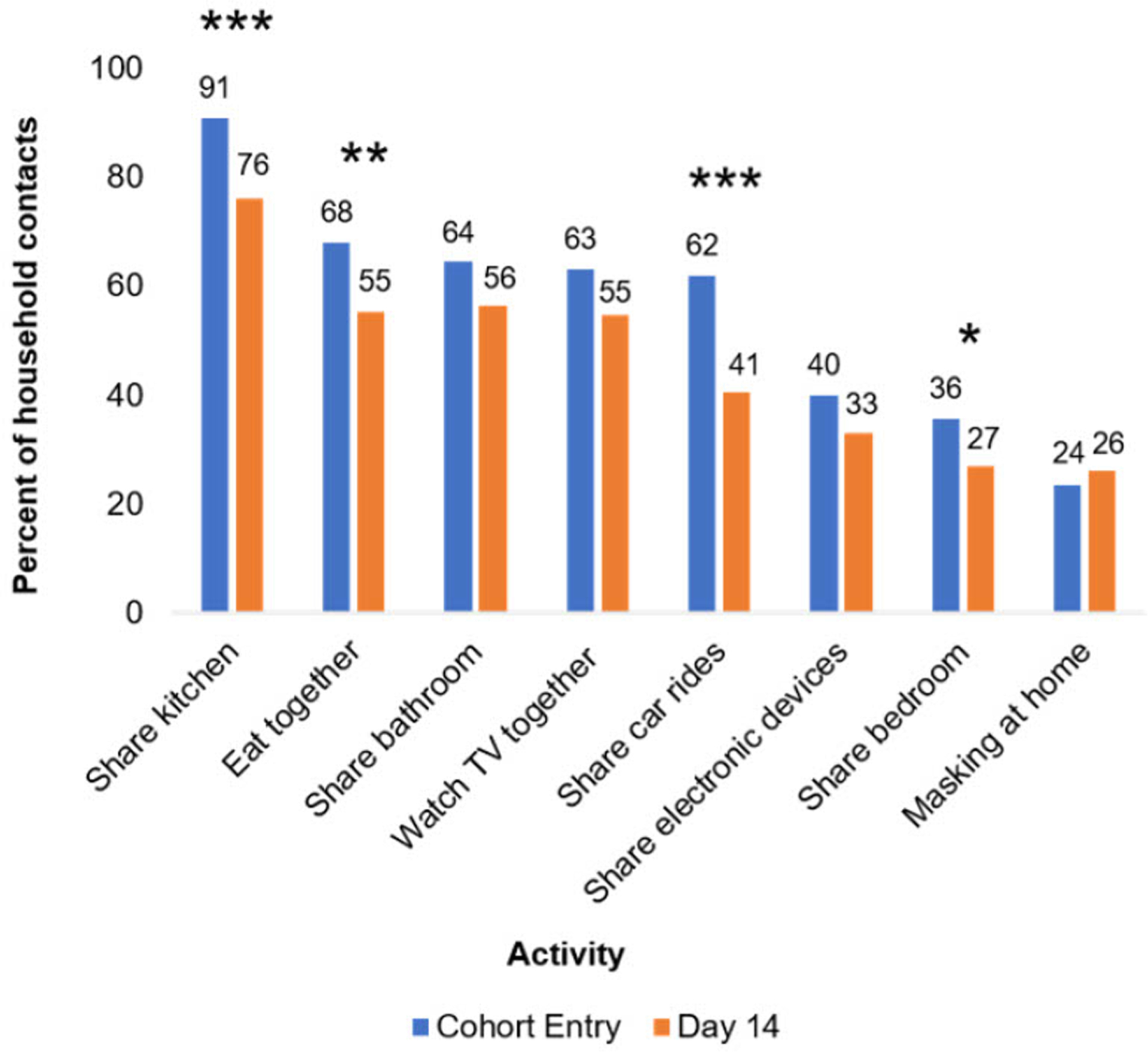
Changes in household contact behaviors from cohort entry to day 14. Entry encompasses the 7 days prior to cohort entry plus the day of enrollment. Day 14 encompasses days 7–14 of participation in the cohort. Participants with non-missing data at both cohort entry and day 14 were included in analysis. Prevalence of behaviors at each time point listed above bars. *p ≤ 0.05, **p ≤ 0.01, and ***p ≤ 0.001. *p*-Values were calculated using Yang’s test for changes from day 0 to 14 on complete cases (Supplementary Table S1). Twenty-four and 74 participants were missing “masking at home” responses at day 0 and 14 respectively, and 41 participants were missing responses for all other variables.

We first assessed changes in household contact behavior from cohort entry to day 14 (Figure 1 and Supplementary Table S1). Using the Obuchowski test for changes in paired binary data with Yang’s modification, we found that several space-sharing behaviors declined from cohort entry to day 14, including the proportion of household contacts who shared a bedroom (36% vs. 27%, *p* ≤ 0.02) or kitchen (91% vs. 76%, *p* ≤ 0.003) with the index case. The proportions who ate with the index case (68% vs. 55%, *p* ≤ 0.02) or rode in a car with the index case (62% vs. 41%, *p* ≤ 0.001) also declined. Still, most contacts shared a kitchen (76%) or bathroom (56%) with the index case and ate or watched TV with (55% each) the index case between days 7 and 14. Despite the prevalence of sharing indoor spaces, only 24% and 26% of household contacts reported that they ever masked at home at cohort entry and day 14, respectively (Figure 1 and Supplementary Table S1).

We also assessed individual and household-level factors associated with (1) ever masking at home and (2) ever sharing a bedroom with the index case between days 7 and 14. Intrahousehold correlation (ICC) was high for the masking variable (ICC = 0.66, 95% CI 0.51–0.79) but low for the bedroom variable (0.10, 95% CI 0.01–0.64). Of 204 household contacts, 74 (36%) were missing masking data and 41 of 204 (20%) household contacts were missing bedroom data among days 7–14.

**FIGURE 2:**
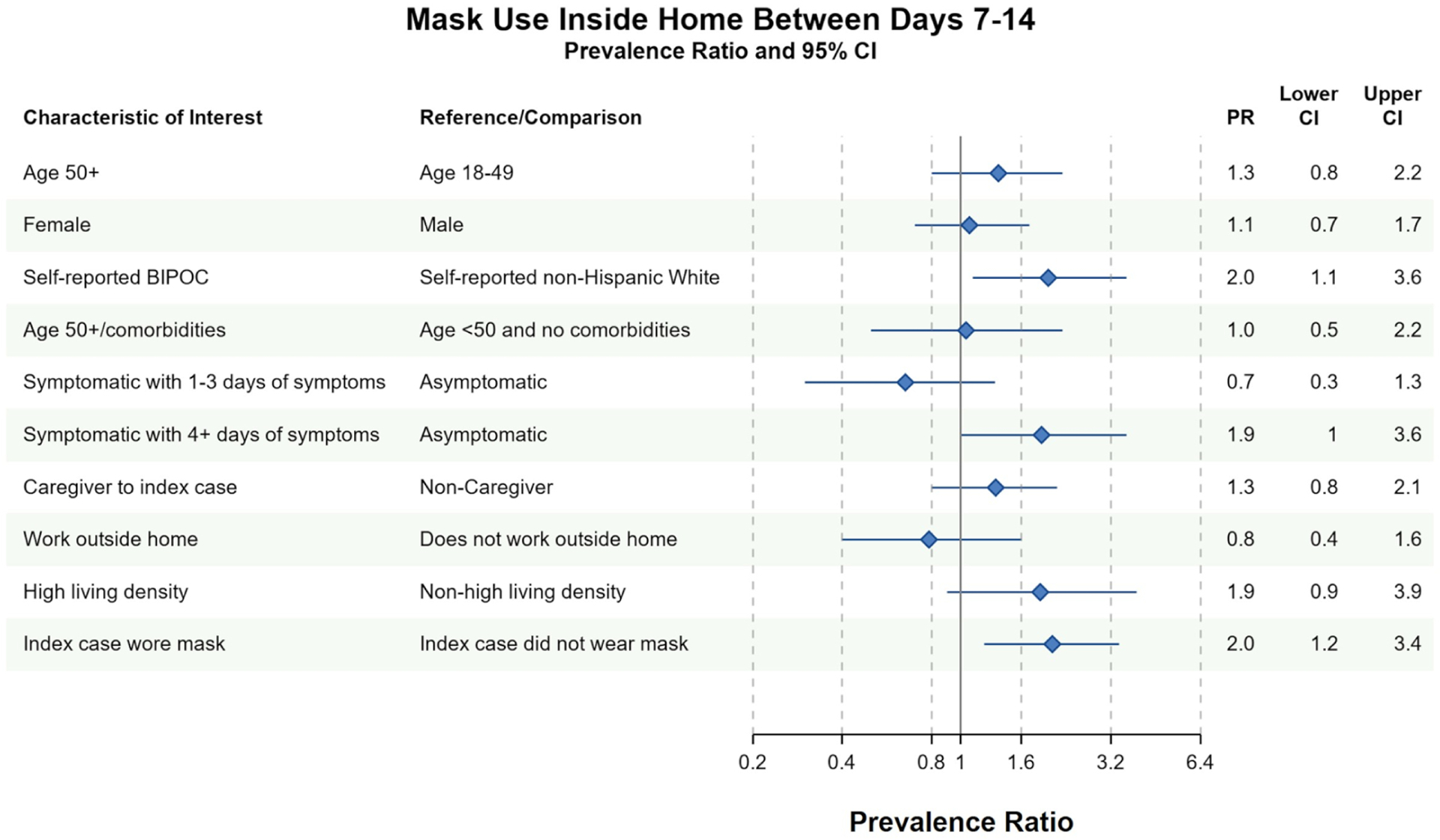
Bivariate complete case analyses of factors associated with wearing a mask at home at any time between days 7–14 of cohort participation. Dots (PR) and solid lines (95% CI) display the complete case analyses. PR and 95% CI are displayed on the natural log scale. Vertical solid line denotes the null value of the PR. *X*-axis labels correspond to the PR values. Sample sizes and prevalence estimates are shown in Supplementary Table S4. BIPOC, Black, Indigenous, People of Color; CI, confidence interval; PR, prevalence ratio. All ages included in PRs with the exception of the first PR.

Household contacts who self-identified as BIPOC were more likely to report masking between days 7–14 than White, non-Hispanic contacts [prevalence ratio (PR) = 2.0, 95% CI 1.1–3.6]. MI did not change the strength of this association (PR = 2.0, 95% CI 1.1–3.8). Household contacts who observed the index case masking between days 7 and 14 were also more likely to mask at home (PR = 2.0, 95% CI 1.2–3.4). This association largely persisted in the MI analysis (PR = 2.0, 95% CI 0.9–4.2) (Figure 2 and Supplementary Figure S2). Contacts with longer symptom duration were also more likely to mask at home in complete case analyses (PR = 1.9, 95% CI 1.0–3.6), although this relationship did not persist in MI (PR = 1.1, 95% CI 0.6–2.0).

**FIGURE 3:**
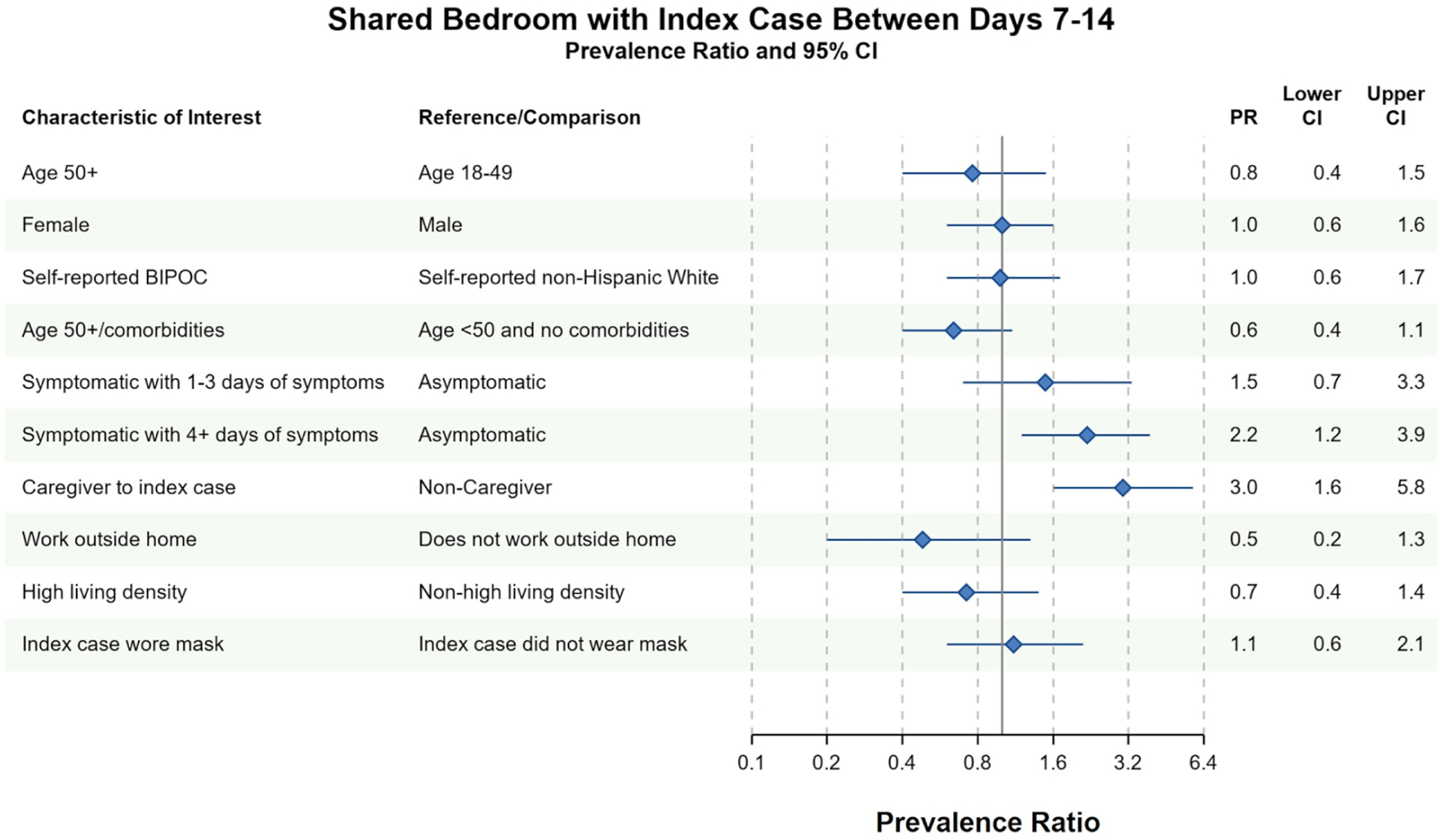
Bivariate complete case analysis of factors associated with sharing a bedroom with the index case at any time between days 7–14 of cohort participation. Dots (PR) and solid lines (95% CI) display the complete case analyses. PR and 95% CI are displayed on the natural log scale. Vertical solid line denotes the null value of the PR. *X*-axis labels correspond to the PR values. Sample sizes and prevalence estimates are shown in Supplementary Table S4. BIPOC, Black, Indigenous, People of Color; CI, confidence interval; PR, prevalence ratio. Table denotes the PR, lower 95% CI and upper 95% CI. All ages included in PRs with the exception of the first PR.

Different factors predicted whether household contacts shared a bedroom with the index case between days 7 and 14. In both complete case and imputed analyses, household contacts were more likely to have shared a bedroom with the index case if they (1) reported 4 or more days of symptoms between days 7 and 14 or (2) identified as the primary caregiver to the index case between days 7 and 14 (Figure 3 and Supplementary Figure S3). Conversely, household contacts at increased risk of severe COVID-19 infection avoided sharing a bedroom with the index case in complete case (PR = 0.6, 95% CI 0.4–1.1) and imputed analyses (PR = 0.7, 95% CI 0.4–1.1). There was no evidence of an association between household contact race/ethnicity and bedroom-sharing in complete case nor imputed sensitivity analyses (PR = 1.0, 95% CI 0.6–1.7). Similarly, index-masking behavior was not associated with household contacts sharing a bedroom with the index case in neither complete case (PR = 1.1, 95% CI 0.6–2.1) nor imputed analyses (PR = 1.2, 95% CI 0.7–2.0) (Figure 3 and Supplementary Figure S3).

Lastly, given the associations between race/ethnicity and masking, and living density and masking, we sought to determine whether living density differed among BIPOC and White, non-Hispanic participants who masked versus those who did not. Among the study participants, BIPOC were more likely than White non-Hispanic individuals to live in a high-density-household (Supplementary Tables S2, S3). Among BIPOC household contacts, the likelihood of in-home masking was similar for those in a high living density household versus a lower density household in both complete case (PR = 1.3, 95% CI 0.5–3.1) and MI (PR = 1.2, 95% CI 0.6–2.5). The association between living in a high-density household and masking was attenuated toward the null when adjusted for BIPOC race/ ethnicity in both complete case (PR = 1.3, 95% CI 0.6–3.0) and MI analysis (PR = 1.2, 95% CI 0.6–2.5).

## 4. Discussion

We prospectively examined associations between household and individual-level factors and transmission-modifying behaviors in households with active COVID-19 infections in a racially and ethnically diverse sample of North Carolina residents. Throughout the 14 days of observation, most household contacts reported not masking inside the home at any time. Nonetheless, we found that household contacts of color and contacts who observed the index case masking were much more likely to mask.

Throughout the study period, over 50% of household contacts continued to share kitchen space, share a bathroom, eat meals, and watch TV with the index case. Our findings suggest that changing behaviors constrained by space and resources, such as sharing bathrooms and kitchens, may be difficult for households. Masking, alternatively, is an inexpensive intervention accessible to most people. Targeted demographic groups, such as White, non-Hispanic households, could be encouraged to mask more frequently, and encouraging infected or symptomatic individuals to mask at home may help convince other household members to also mask.

Unlike other studies that measured the household transmission of SARS-CoV-2 (8) or behavioral interventions at the community level, our study prospectively measured the behaviors of household contacts after an initial household infection was identified. In early 2022, Baker and colleagues reported a retrospective analysis of behaviors of household members exposed to SARS-CoV-2 in Chicago, Milwaukee, Connecticut, and Utah in the winter of 2021–2022. However, their analysis did not identify demographic characteristics of household contacts who engaged in behaviors such as masking, instead focusing on behaviors associated with transmission (8), as did our primary analysis of CO-HOST participants (4).

Other studies evaluated the attitudes and beliefs toward masking and isolating from family members if exposed to SARS-CoV-2, although they did not prospectively measure household contacts’ behavior. In the United Kingdom, adults were asked whether they would self-isolate away from home if infected or exposed if they were provided appropriate accommodations at no cost (23). Among the participants who noted that they would not be able to isolate from family members at home if infected, 56% noted that they would definitely or probably accept accommodations if offered to them. Many of these individuals cited household size and the number of household residents as barriers to isolating within the home. In interviews, low-income participants and participants from racial and ethnic minority communities highlighted the elevated risk of exposure they faced at work as a driving force to accept free accommodations outside the home.

In our study, similar concerns may also explain why BIPOC household contacts and contacts living in high-density households were more likely to have masked at home, although we did not ask household contacts why they masked. While there was no clear association between living density and masking after adjusting for BIPOC race/ethnicity, BIPOC participants were overall more likely to live in high-density households. It is plausible that participants of color within our study understandably had a greater concern of contracting and surviving infection, given highly publicized racial disparities in COVID-19 infection and fatalities as early as Spring 2020 (24, 25). These concerns could have motivated BIPOC participants to mask at home, given the structural barriers to isolation, such as high living density, and the lack of government-sponsored accommodations for exposed or infected individuals to isolate in North Carolina and much of the USA.

In our study, household contacts who observed their index case masking at home were themselves more likely to mask. Household members may share similar beliefs around the efficacy of masking, the science of SARS-CoV-2 transmission, and the severity of the virus infection (8). In a “Prisoners’ Dilemma” simulation of mask-wearing among adults in the USA, participants who chose not to wear masks were more likely to cooperate with non-mask-wearers than mask-wearers, suggesting that in-group dynamics and social identity play a role in the decision to mask (26). Together, findings from our study and the Prisoners’ Dilemma simulation suggest that campaigns encouraging infected and symptomatic individuals to mask at home may encourage their household members to mask as well. In-home masking may be particularly feasible for asymptomatic positive individuals, whereas some individuals with respiratory symptoms or young children may find it difficult to mask consistently. Moreover, masking is not recommended during sleep (27), underscoring the importance of having the ability to sleep in a separate bedroom from infected individuals.

Our analyses of bedroom-sharing identified that household contacts who worked outside the home in the previous week or who had risk factors for severe COVID-19 were less likely to share a bedroom with the index case, and that individuals with 4 or more days of symptoms were more likely to have shared a bedroom with the index case. In our cohort, secondary infections were more likely among household contacts who shared a bedroom with the index case (4). Our findings suggest that household contacts who faced steeper consequences of infection (e.g., missed days of work, higher risk of severe COVID-19) opted not to share a bedroom with the index case where possible.

The strengths of our study include the longitudinal design, a racially diverse sample, the use of multiple imputation to account for missing data, and the unique scope of our question on structural household factors associated with behaviors that affect household transmission. Our study included more Hispanic/ Latinx-identifying participants compared to North Carolina at large (10.7%) and compared to the counties in which our participants resided (9.6%–20.7%), including the counties of Durham, Orange, Alamance, Chatham, Wake, Lee, and Guilford (4). While the proportion of Black, non-Hispanic participants in our study was lower than the counties of residence of our participants (10.4%–34.1%), the proportion of White, non-Hispanic participants in our study fell within the range of participants’ counties (42.9%–71.4%) (28). Our results also reflect the racial/ethnic breakdown of the USA at large recorded in the 2020 Census, in which 57.8% of the participants identified as non-Hispanic White, compared to 52.5% in our study (29). This supports the generalizability of our study findings to North Carolina.

Our study nonetheless is limited by sample size, and possible reporting bias by the respondents, given the extent of missing data in the study. Moreover, the phrasing of the binary masking variable did not measure masking frequency at home. Participants answered “yes” to the masking variable if they recalled having ever masked at home between days 7 and 14. In addition, while the study was prospective, behavioral outcomes were ascertained only at two timepoints. We were also limited by the amount of information we were able to collect; important variables such as socioeconomic status (SES), health insurance status, and participation in public social safety net programs was not measured, and thus remain a potential source of unmeasured confounding. Nonetheless, we did collect information on other correlates of SES, such as education and square footage of the home. Lastly, recall bias and social desirability bias could weaken the validity of our results.

We investigated the predictors of physical distancing behaviors among household contacts exposed to SARS-CoV-2 in a period of high susceptibility to COVID-19 infection. Vaccines were not available, and most people were unexposed (30). Today, widespread vaccination and therapeutics (e.g., nirmatrelvir and ritonavir) have reduced the risk of severe disease (31, 32). However, the risk of household transmission and long COVID-19 complications remains considerable (31, 33, 34), given increased transmissibility and immune escape among new variants leading to an increase in breakthrough infections and reinfections (35). In the ongoing phase of the COVID-19 pandemic, our findings support additional congressional funding to continue the Biden administration’s SARS-CoV-2 at-home rapid antigen test distribution program to any American household. We also encourage the administration to distribute N95 masks at the federal level, given the prohibitive cost for large households. Virtually no published studies have assessed the attitudes and motivations for masking and isolating among infected and exposed household members in the USA, a large and diverse country where many communities likely have their own beliefs and barriers around masking and isolation at home. Nonetheless, we have sufficient information to justify public health campaigns increasing the feasibility and social desirability of masking and isolating among exposed household members where possible, and the need for government and private-sector support of outside accommodations where isolation and masking are impossible.

## Supplementary Material

Supplementary Data

## Figures and Tables

**TABLE 1:** Characteristics of household contacts at cohort entry.

Variable	Overall	BIPOC^a^	White, non-Hispanic
Household-level characteristics	*N* = 100 households	*N* = 54 households	*N* = 46 households
**Number of household members in each household**			
2 people	27 (27.0)	14 (25.9)	13 (28.3)
3 people	23 (23.0)	11 (20.4)	12 (26.1)
4 people	22 (22.0)	9 (16.7)	13 (28.3)
5 or more people	28 (28.0)	20 (37.0)	8 (17.4)
**Number of rooms in house** ^b^			
2 or fewer rooms	10 (10.0)	7 (13.0)	3 (6.5)
3–5 rooms	43 (43.0)	31 (57.4)	12 (26.1)
6 or more rooms	47 (47.0)	16 (29.6)	31 (67.4)
**Number of square feet in house**			
<500 sq feet (<46.5 sq m)	3 (3.0)	2 (3.7)	1 (2.2)
500–1,000 sq feet (46.5–93 sq m)	17 (17.0)	12 (22.2)	5 (10.9)
1,000–2,000 sq feet (93–186 sq m)	33 (33.0)	19 (35.2)	14 (30.4)
>2,000 sq feet (>186 sq m)	42 (42.0)	16 (29.6)	26 (56.5)
Unknown	5 (5.0)	5 (9.3)	0 (0.0)
**Household with high living density** ^c^			
Yes	23 (23.0)	20 (37.0)	3 (6.5)
No	77 (77.0)	34 (63.0)	43 (93.5)
% **of household members (including index cases) with COVID-like symptoms by day 7**^d,e^			
<50	14 (22.6)	7 (21.2)	7 (24.1)
50-<100	15 (24.2)	8 (24.2)	7 (24.1)
100 (all members)	33 (53.2)	18 (54.6)	15 (51.7)
Missing	4	2	
Individual-level characteristics	*N* = 204 participants	*N* = 97 participants	*N* = 107 participants
**Age**			
1–4 years	11 (5.4)	6 (6.2)	5 (4.7)
5–12 years	35 (17.2)	17 (17.5)	18 (16.8)
13–17 years	24 (11.8)	12 (12.3)	12 (11.2)
18–24 years	25 (12.3)	11 (11.3)	14 (13.1)
25–49 years	67 (32.8)	35 (36.1)	32 (29.9)
50–64 years	30 (14.7)	10 (10.3)	20 (18.7)
>65 years	12 (5.9)	6 (6.2)	6 (5.6)
**Current sex**			
Male	98 (48.9)	46 (47.4)	52 (48.6)
Female	106 (52.0)	51 (52.6)	55 (51.4)
**Race/ethnicity**			
White, non-Hispanic	107 (52.5)		
Hispanic/Latinx	70 (34.3)		
Black, non-Hispanic	18 (8.8)		
Other Race/Unknown Race^f^, non-Hispanic	9 (4.4)		
**Education**			
Children under 18	70 (35.0)	35 (37.6)	35 (32.7)
Adult, high-school or less	63 (31.5)	41 (44.1)	22 (20.6)
College degree	38 (19.0)	11 (11.8)	27 (25.2)
Graduate degree	29 (14.5)	6 (6.5)	23 (21.5)
Missing	4		
**Any comorbidities** ^g^			
Yes	71 (35.5)	38 (40.0)	33 (31.4)
No	129 (64.5)	57 (60.0)	72 (68.6)
Missing	4	2	2
**BMI ≥30** ^h^			
Yes	46 (31.9)	27 (46.6)	19 (22.1)
No	98 (68.1)	31 (53.5)	67 (77.9)
Missing	18	17	1
**COVID-19 like symptoms in past 7 days** ^d^			
Yes	109 (54.0)	49 (51.6)	60 (56.1)
No	93 (46.0)	46 (48.4)	47 (43.9)
Missing	2	2	0
**Relationship to primary infected case**			
Partner	58 (28.7)	21 (22.1)	37 (34.6)
Child	68 (33.7)	32 (33.7)	36 (33.6)
Sibling, including in-laws	19 (9.4)	13 (13.7)	6 (5.6)
Parent, including in-laws	35 (17.3)	19 (20.0)	16 (15.0)
Roommate/friend	15 (7.4)	7 (7.4)	8 (7.5)
Other relative/other^i^	7 (3.5)	3 (3.2)	4 (3.7)
Missing	2	2	
**Caregiver to primary infected case** ^h^			
Yes	58 (38.7)	21 (31.8)	37 (44.1)
No	92 (61.3)	45 (68.2)	47 (56.0)
Missing	12	9	3
**Index case ever wore mask in the home past 7 days**			
Yes	153 (80.1)	79 (90.8)	74 (71.2)
No	38 (19.9)	8 (9.2)	30 (28.9)
Missing	13	10	3
**Live with someone under 18** ^j^			
Yes	72 (50.4)	31 (49.2)	41 (51.3)
No	71 (49.7)	32 (50.8)	39 (48.8)
**Association to healthcare facility** ^j,k^			
Works in a healthcare facility	7 (5.0)	2 (3.2)	5 (6.3)
Household includes someone who works in a healthcare facility	18 (12.8)	6 (9.7)	12 (15.2)
Neither	116 (82.3)	54 (87.1)	62 (78.5)
Missing	2	1	1

a Includes non-Hispanic Black, Hispanic/Latinx of any race, Asian American and Pacific Islander, Native American and Alaska Native, Other race, and Mixed race. Households were considered to be BIPOC if at least one CO-HOST participant (index case or household contact) self-identified as BIPOC.

b Including bedrooms, kitchen, and common rooms, but not bathrooms or garage.

c More than three persons occupying <6 rooms, including bedrooms, kitchen, and common rooms, but not bathrooms or garage.

d Symptoms assessed in daily symptom surveys included fever, chills, muscle aches, runny nose, sore throat, loss of taste or smell, cough, shortness of breath, chest pain, wheezing, nausea, diarrhea, headache, and abdominal pain.

e *N* = 66 households in which every member of the household was enrolled.

f Self-identified races include American Indian or Alaska Native, Asian, Native Hawaiian or Other Pacific Islander, Other Race, Unknown Race, or Refusal.

g Comorbidities include HIV, chronic lung disease (e.g., emphysema, chronic obstructive pulmonary disease), asthma, daily smoking, heart disease (e.g., previous heart attack, heart failure, stents), morbid obesity (>100 pounds over ideal weight), diabetes, high blood pressure, chronic kidney disease, chronic liver disease, weak immune system due to disease or medication, and recent (within past 2 weeks) or current pregnancy.

h Includes participants ages 12 and over.

i Other relationships include sibling, parent, other relative, roommate and friend/non-roommate.

j *N* = 143 household contacts who live in households in which every member of the household was enrolled.

k Worked in a healthcare facility in the past 14 days or live with someone who worked in a healthcare facility in last 14 days.

## Data Availability

The raw data supporting the conclusions of this article will be made available by the authors, without undue reservation.
