# Supplementary material for: Transmission prevention behaviors in US households with SARS-CoV-2 cases in 2020
Source: Front Epidemiol. Author manuscript; Available in PMC 2024 Feb 23. (PMC10888502; doi:10.3389/fepid.2023.1160214)
Supplement: Supplementary Data [file NIHMS1917118-supplement-Supplementary_Data.docx]

Supplementary Material

Transmission prevention behaviors in US households with SARS-CoV-2 cases in 2020

Rebecca J Rubinstein*, Wenwen Mei, Caitlin A. Cassidy, Gabrielle Streeter, Christopher Basham, Carla Cerami, Feng-Chang Lin, Jessica T. Lin, Katie R. Mollan

*** Correspondence:** Corresponding Author: [Rebecca_rubinstein@med.unc.edu](mailto:Rebecca_rubinstein@med.unc.edu)

# Supplementary Data

Supplementary Material should be uploaded separately on submission. Please include any supplementary data, figures and/or tables.

Supplementary material is not typeset so please ensure that all information is clearly presented, the appropriate caption is included in the file and not in the manuscript, and that the style conforms to the rest of the article.

# Supplementary Figures and Tables

For more information on Supplementary Material and for details on the different file types accepted, please see [here](https://www.frontiersin.org/guidelines/author-guidelines#supplementary-material).

## Supplementary Figures


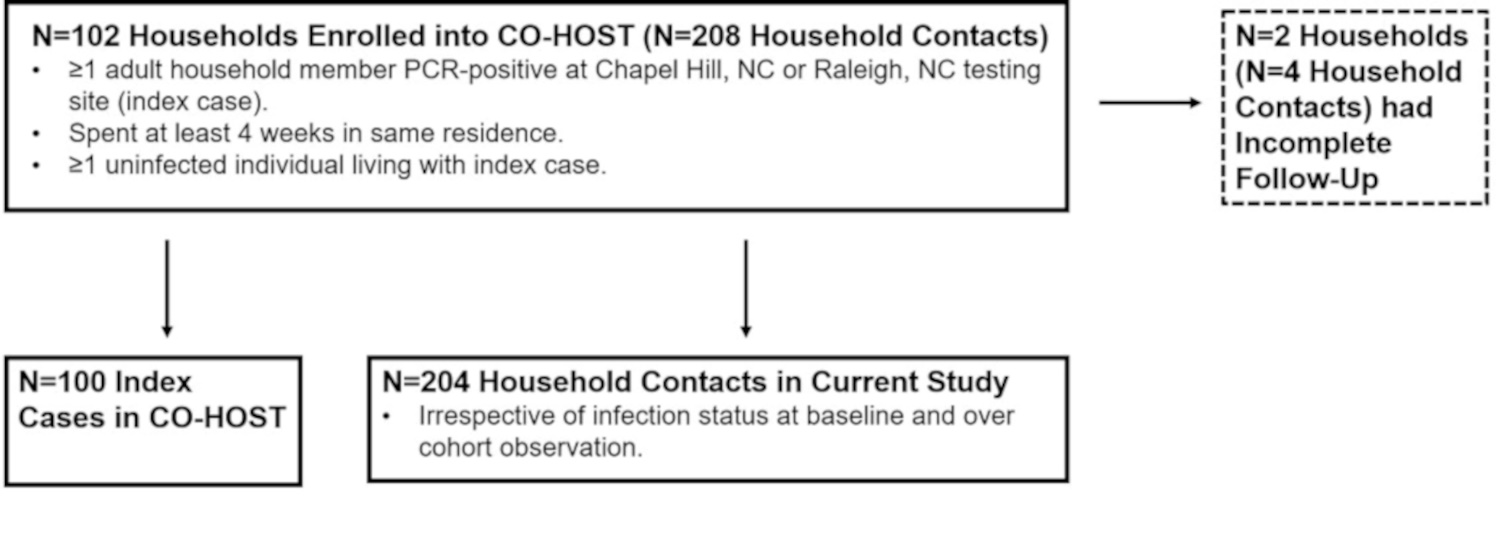


**Supplementary Figure 1.** **Inclusion diagram**. CO-HOST refers to the parent study from which this secondary analysis is derived. 2 households consisting of 2 index cases and 4 household contacts were unable to be included in this study due to insufficient follow up. This study utilized the 204 remaining household contacts.

**
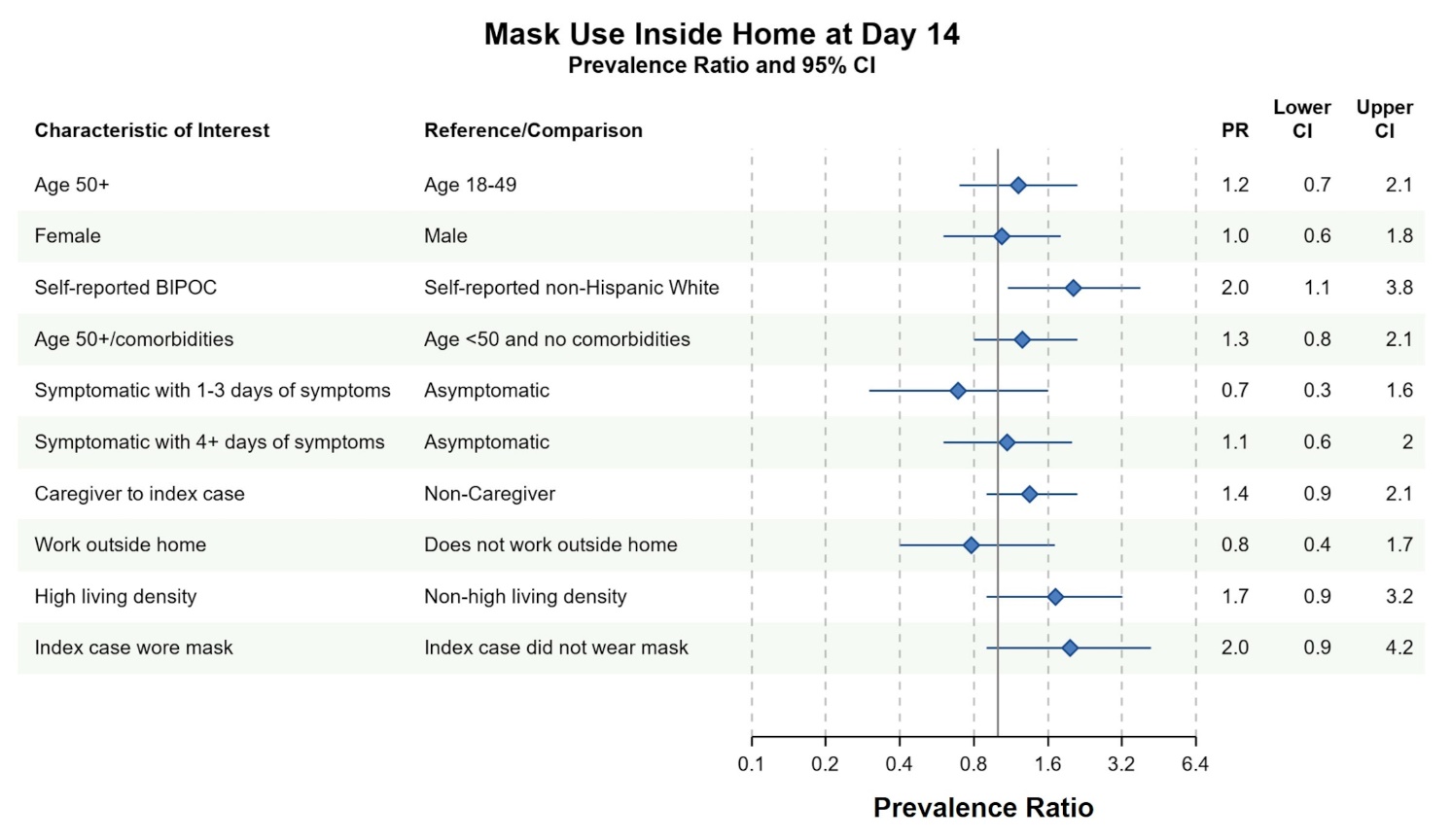
**

**Supplementary Figure 2. Bivariate sensitivity analyses of factors associated with using a mask at home at any time at Day 14 of cohort participation**. Solid dots (PR) and solid lines (95% CI) display imputed estimates using chained multiple imputation for clustered data. PR and 95% CI graphed on the natural log scale. Vertical solid line denotes the null value of the PR (PR=1.0) on the exponentiated scale. X-axis labels correspond to the exponentiated scale. BIPOC=Black, Indigenous, People of Color. Table denotes the PR, lower 95% CI and upper 95% CI.


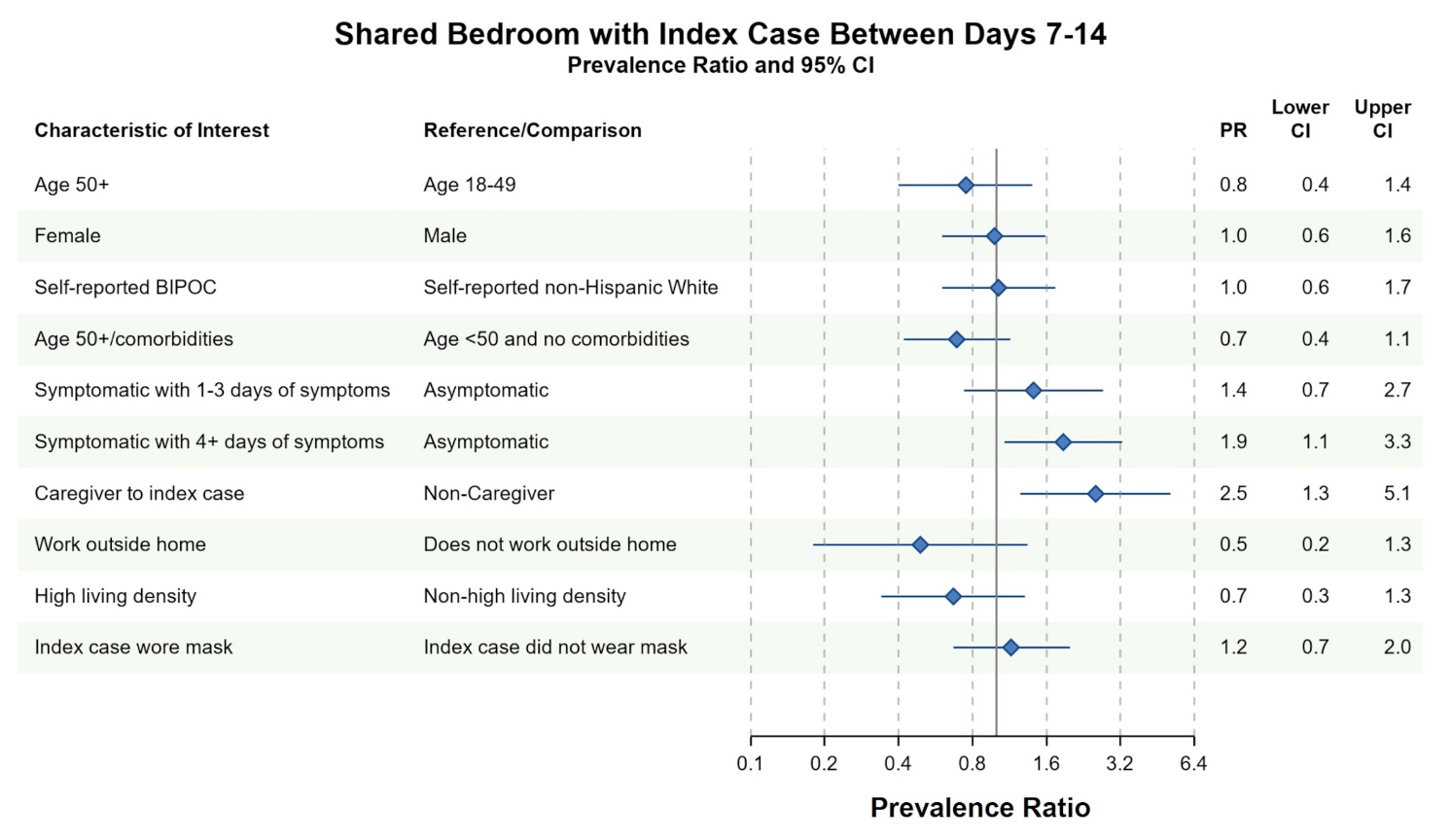


**Supplementary Figure 3. Bivariate sensitivity analyses of factors associated with sharing a bedroom with the index case at any time between Days 7-14 of cohort participation**. Solid dots (PR) and solid lines (95% CI) display imputed estimates using chained multiple imputation for clustered data. PR and 95% CI graphed on transformed natural log scale. Vertical solid line denotes the null value of the PR (PR=1.0) on the exponentiated scale. X-axis labels correspond to the exponentiated scale. BIPOC=Black, Indigenous, People of Color. Table denotes the PR, lower 95% CI and upper 95% CI.

**Supplementary Table 1. Number and proportion of household contacts engaging in reported behaviors at study entry and Day 14.** P-values were calculated using Yang’s test for changes between Day 0-14 on complete cases. 85 participants were missing ‘masking at home’ responses and 119 were evaluable. 41 participants were missing responses for all other variables and 163 participants were evaluable. See Figure 1 in main text.

| **Behavior** | **N (%) Household Contacts Reporting at Cohort Entry** | **N (%) Household Contacts Reporting at Day 14** | **P-value** |
| --- | --- | --- | --- |
|  |  |  |  |
| Share kitchen | 148 (91) | 124 (76) | 0.003 |
| Eat together | 111 (68) | 90 (55) | 0.013 |
| Share bathroom | 105 (65) | 92 (56) | 0.066 |
| Watch TV together | 103 (63) | 89 (55) | 0.068 |
| Share car rides | 101 (62) | 66 (41) | <0.000 |
| Share electronic devices | 65 (40) | 54 (33) | 0.092 |
| Share bedroom | 58 (36) | 44 (27) | 0.015 |
| Masking at home | 28 (24) | 31 (26) | 0.614 |

**Supplementary Table 2. Number (row %) of household contacts living in high density households among self-reported race/ethnicity and masking behavior.**

| **Mask Use Inside Home Between Days 7-14** | **Race/Ethnicity** | **High Living Density** | | |
| --- | --- | --- | --- | --- |
|  |  | **Yes** | **No** | **Total** |
| **Yes** | **Non-Hispanic White** | 2 (13) | 13 (87) | 15 (100) |
|  | **BIPOC** | 12 (57) | 9 (43) | 21 (100) |
| **No** | **Non-Hispanic White** | 7 (10) | 61 (90) | 68 (100) |
|  | **BIPOC** | 14 (54) | 12 (46) | 26 (100) |

**Supplementary Table 3. Number (%) of household contacts living in high density households among self-reported race/ethnicity and bedroom sharing with index case.**

| **Shared Bedroom with Index Case Between Days 7-14** | **Race/Ethnicity** | **High Living Density** | | |
| --- | --- | --- | --- | --- |
|  |  | **Yes** | **No** | **Total** |
| **Yes** | **Non-Hispanic White** | 1 (4) | 27 (96) | 28 (100) |
|  | **BIPOC** | 8 (53) | 7 (47) | 15 (100) |
| **No** | **Non-Hispanic White** | 10 (14) | 64 (87) | 74 (100) |
|  | **BIPOC** | 21 (47) | 24 (53) | 45 (100) |

**Supplementary Table 4. Frequency and prevalence of household contacts who reported ever masking or ever sharing a bedroom with the index case between Days 7-14 of cohort participation.**

| **Outcome:** | **Mask Use Inside the Home** | | **Sharing a Bedroom** | |
| --- | --- | --- | --- | --- |
| **Household-Contact Characteristics** | **N** | **Prevalence** | **N** | **Prevalence** |
| Age (restricted to 18 and older) |  |  |  |  |
| *18-49 (ref)* | 16 | 0.26 | 24 | 0.33 |
| *50+* | 9 | 0.36 | 8 | 0.24 |
| Sex |  |  |  |  |
| *Male (ref)* | 15 | 0.26 | 20 | 0.27 |
| *Female* | 21 | 0.29 | 24 | 0.27 |
| Race/Ethnicity |  |  |  |  |
| *Non-Hispanic White* | 15 | 0.18 | 28 | 0.27 |
| *Participants of Color* | 21 | 0.45 | 16 | 0.26 |
| Aged 50 or older or any comorbidities^a^ |  |  |  |  |
| *No* | 12 | 0.21 | 24 | 0.35 |
| *Yes* | 24 | 0.35 | 19 | 0.22 |
| *Missing* | 6 |  | 6 |  |
| Duration of COVID-19 Symptoms^b^ |  |  |  |  |
| *No symptoms* | 20 | 0.18 | 18 | 0.16 |
| *1-3 days* | 5 | 0.16 | 8 | 0.26 |
| *4 or more days* | 7 | 0.22 | 12 | 0.38 |
| *Missing* | 28 |  | 28 |  |
| Caregiver to index case^b^ (restricted to 18 and older) |  |  |  |  |
| *No* | 10 | 0.24 | 7 | 0.15 |
| *Yes* | 15 | 0.34 | 23 | 0.47 |
| *Missing* | 0 |  | 2 |  |
| Work outside home on most days^b^(restricted to 18 and older) |  |  |  |  |
| *No* | 22 | 0.32 | 29 | 0.33 |
| *Yes* | 3 | 0.17 | 3 | 0.16 |
| Live in household with high living density |  |  |  |  |
| *No* | 22 | 0.16 | 35 | 0.29 |
| *Yes* | 14 | 0.22 | 9 | 0.21 |
| *Missing* | 74 |  | 0 |  |
| Index case wore mask^b^ (include all age groups) |  |  |  |  |
| *No* | 4 | 0.09 | 15 | 0.25 |
| *Yes* | 32 | 0.37 | 27 | 0.27 |
| *Missing* | 0 |  | 3 |  |

a We considered individuals aged 50 or older or those with at least one comorbidity to be at higher risk for severe COVID-19 infection. If the household contact was the only member of their household with higher risk for severe COVID-19 infection, they were placed in the reference group. Household contacts who resided in households in which some members were not enrolled in COHOST, and where there were no known household members over aged 50 or with comorbidities, were coded as missing.

b Between days 7-14 of cohort observation
